# Transformers for Urban Sound Classification—A Comprehensive Performance Evaluation

**DOI:** 10.3390/s22228874

**Published:** 2022-11-16

**Authors:** Ana Filipa Rodrigues Nogueira, Hugo S. Oliveira, José J. M. Machado, João Manuel R. S. Tavares

**Affiliations:** 1 Faculdade de Ciências, Universidade do Porto, Rua do Campo Alegre 1021 1055, 4169-007 Porto, Portugal; 2 Faculdade de Engenharia, Universidade do Porto, Rua Dr. Roberto Frias, s/n, 4200-465 Porto, Portugal; 3 Departamento de Engenharia Mecânica, Faculdade de Engenharia, Universidade do Porto, Rua Dr. Roberto Frias, s/n, 4200-465 Porto, Portugal

**Keywords:** urban sounds’ classification, deep learning, convolutional neural network, data augmentation, Adam optimizer

## Abstract

Many relevant sound events occur in urban scenarios, and robust classification models are required to identify abnormal and relevant events correctly. These models need to identify such events within valuable time, being effective and prompt. It is also essential to determine for how much time these events prevail. This article presents an extensive analysis developed to identify the best-performing model to successfully classify a broad set of sound events occurring in urban scenarios. Analysis and modelling of Transformer models were performed using available public datasets with different sets of sound classes. The Transformer models’ performance was compared to the one achieved by the baseline model and end-to-end convolutional models. Furthermore, the benefits of using pre-training from image and sound domains and data augmentation techniques were identified. Additionally, complementary methods that have been used to improve the models’ performance and good practices to obtain robust sound classification models were investigated. After an extensive evaluation, it was found that the most promising results were obtained by employing a Transformer model using a novel Adam optimizer with weight decay and transfer learning from the audio domain by reusing the weights from AudioSet, which led to an accuracy score of 89.8% for the UrbanSound8K dataset, 95.8% for the ESC-50 dataset, and 99% for the ESC-10 dataset, respectively.

## 1. Introduction

Cities are consolidating their position as the central structure in human organizations worldwide, and it is expected that, by 2050, 80% of the world’s population will live in urban environments. This rapid trend of urbanization creates huge development opportunities to improve citizens’ lives. Smart Cities take advantage of these opportunities by providing new and disruptive city-wide services using sensing architectures deployed in the cities to increase the quality of their dwellers [1].

Sound is an important source of information that can serve as an alternative or complement to various forms of environmental sensing, such as imaging and video cameras. Therefore, Smart Cities can take advantage of urban sound characterization due to the possibility of being applied in diverse tasks such as noise pollution mitigation, security, monitoring, context-aware computing, autonomous vehicle guidance, and surveillance, just to name a few [1,2]. However, urban environments contain unlimited and co-occurring sound events, which poses a difficulty due to the need to recognize complex acoustic scenes from daily life. Therefore, rapid events that require immediate action often go unnoticed by city authorities. Thus, the scientific community has been developing different computational algorithms to acquire, analyse, and classify urban sounds automatically. Nonetheless, the combination of multiple classes, abnormal noise conditions, and the multiplicity of sound sources are still limitations for efficiently completing the task [1,3,4,5].

Initially, researchers focused on using handcrafted features to find which would provide more discriminating characteristics to classify different sound events [6,7,8,9]. Later, with the growth of Deep-Learning (DL)-based approaches, deep features have shown a higher capacity to give more relevant information than handcrafted features. Thus, Convolutional Neural Network (CNN)s have been proposed due to their ability to learn local and high-level features on the image space [6,8]. Furthermore, most current approaches explore the use of pre-trained CNNs, by redefining the last layers to address the sound classification problem [5,10]. The downside is the inability to adequately capture the long time dependencies in an audio clip [11]. Other works proposed solutions based on CNNs supported by Recurrent Neural Network (RNN)s [11,12] to be able to save historical information, allowing taking into account long temporal information [11], and by exploring Long Short-Term Memory (LSTM) models [4,12] to prevent the vanishing gradient problem. However, these models are not able to perform calculations in parallel. More recently, attention mechanisms have been incorporated to focus on semantically important parts of the sound under study [13,14,15,16,17]. Lately, solutions based on attention models [11,18], particularly on Transformers [18,19,20,21,22], are being explored.

Transformers have a network architecture based exclusively on attention mechanisms. This architecture, because it can make predictions based entirely on attention mechanisms, performs parallel computations and incorporates the global feature context, allowing the achievement of reliable results. These characteristics have made it a very appealing architecture to be explored in various areas such as Natural Language Processing (NLP), computer vision, and more recently, sound-related areas. Nonetheless, to achieve high-accuracy results, Transformers require vast amounts of data. In addition, some new augmentation techniques have been proposed to avoid overfitting, diversify the training process, and solve the scarcity of data [5,23]. However, the optimal architecture for each application has not yet been established, and many problems remain, namely identifying the models performing better in a wide set of audio datasets, the most useful augmentation techniques, and the most challenging sound classes to classify. According to the “no free lunch” theorem (Wolpert and Macready [24]), no optimization algorithm is capable of achieving the best performance for all possible applications, suggesting that, if all optimization techniques were averaged over all potential objective functions, their performance would be equally effective. This optimization possibility also means that the most suitable optimization technique for a particular application allows for obtaining a good model.

Moreover, there is an open question regarding the use of these techniques. This study addressed this question quantitative and qualitatively by evaluating several model architectures on different datasets and determining the most general-purpose architecture. Complementary parameters and data augmentation methods were also analysed to assess the performance in all evaluated sound datasets. Finally, a group of practices was setup to determine the best model for sound classification tasks. With this in mind, this article is organized as follows: Section 2 presents a literature review of recent related works. Section 3 presents the methodology followed in the current study. Section 4 describes the experimental validation and discusses the results of the performed experiments for the sound classification models under study and the corresponding augmentation and transfer learning techniques by establishing comparisons among attention, convolution, and feature-based models. Section 5 discusses the results as an aggregated summary of the findings and presents the conclusions and sketches of future work.

## 2. Related Works

This section identifies major works proposed to tackle the problem of sound classification.

### 2.1. CNN for Audio Classification

Like the image classification problem, a CNN is a natural architecture for audio classification. Therefore, researchers explored the use of this architecture, namely: Salamon and Bello [23], by employing a Deep Convolutional Neural Network (DCNN); Das et al. [3] explored the use of a CNN model with a specific Additive Angular Margin Loss (AAML) and also explored a CNN combined with stacked features such as Mel Frequency Cepstral Coefficients (MFCC) and Chromagram; Mu et al. [6] introduced the Temporal-Frequency Attention-Based Convolutional Neural Network (TFCNN) model, a CNN-based model associated with attention mechanisms, among others.

In data augmentation, noise injection, shifting time, changing pitch, and speed are the most common techniques used to solve the scarcity of labelled data for training. Additionally, Salamon and Bello [23] proposed four different augmenting deformations to apply to the training set: time stretching to slow down or speed up the audio sample, with the pitch remaining unchanged; pitch shifting, where the audio sample’s pitch is raised or lowered while keeping the duration unchanged; dynamic range compression, to compress the dynamic range of the audio using parameterizations from the Dolby E standard and the Icecast online radio streaming server; background noise addition, which is similar to noise injection, but where recordings of background sounds from different scenes are mixed with the audio sample to make models more robust to intermixed sounds.

### 2.2. RNN for Audio Classification

CNNs combined with RNNs are commonly explored to model audio in sound classification problems. Das et al. [4] used an LSTM in combination with spectral features obtained from audio training segments. In the research presented by Das et al. [4], a comparative study between a CNN and an LSTM model using different combinations of spectral features was described.

Other studies were focused on incorporating attention mechanisms to improve the Convolutional Recurrent Neural Network (CRNN) models’ performance, such as the works of Zhang et al. [13,14], Qiao et al. [15]. The study presented by Zhang et al. [13] incorporates temporal attention for reducing the impact of background noise and channel attention mechanisms, aiming to achieve more attention on the filters and allowing for the detection of the essential characteristics of the sounds under study.

Later, Zhang et al. [14] used a frame-level attention mechanism to focus on critical temporal events while reducing the impact of background noise. Moreover, applying the attention mechanism to lower layers helped to preserve lower-level features. Furthermore, employing attention for RNN layers led to the highest accuracy results. On the other hand, the sigmoid function used as a scaling function led to better attention weights than the Softmax function when applying attention at the CNN layers.

To demonstrate the advantages of an attention mechanism, Qiao et al. [15] developed a CRNN model with a temporal-frequency attention mechanism and a CRNN model using sub-spectrogram segmentation-based feature extraction and score level fusion.

Most models employ some degree of data augmentation in the training data, such as pitch shift, time stretch, and pitch shift with time stretch, to improve their performance.

### 2.3. Transformers for Audio Classification

Motivated by the limitations of capturing long-range dependencies, several authors have recently adopted the use of attention mechanisms to address the sound classification problem. For example, Mu et al. [6] proposed a TFCNN that reduces the influence of background noise and irrelevant frequency bands due to the frequency and temporal attention mechanisms. Several hybrid architectures combining Transformers with CNNs are commonly used, for example in the work of Kong et al. [11], being a common standard to exploit the potentialities of both architectures.

Regarding Pure Transformers, Elliott et al. [19], Wyatt et al. [20], and Devlin et al. [25] explored the advantages of Bidirectional-Encoder-Representations-from-Transformers (BERT)-based models, based on the work of Vaswani et al. [26], having as the input a given token summed with the position embeddings, in order to address the sound classification problem at the edge.

Similarly, Gong et al. [9] presented an Audio Spectrogram Transformer (AST) purely based on the attention-based model. Park et al. [21] also explored AST by using a Many-to-Many Audio Spectrogram Transformer (M2M-AST), allowing output sequences with different resolutions for multi-channel audio inputs. To reduce the training complexity, Akbari et al. [18] presented a regularization technique called DropToken in combination with a Video–Audio–Text Transformer (VATT) model, which achieved competitive performance. Koutini et al. [22] also presented a regularization technique named Patchout that randomly erases patch chunks from the input sequence.

Both on Hybrid and Pure Transformers, the model’s performance can be improved while effectively avoiding overfitting through transfer learning, erasing a certain number of frequency bins or time frames, gain change, and random patch spectrograms erasing, to train audio classification Transformer-based models.

## 3. Proposed Approach

To evaluate the impact of several model architectures and complementary methods that have been proposed, extensive experiments were conducted using feature-based models, mainly CNN architectures. Transformers with attention mechanisms were also extensively evaluated to determine the most competitive model for urban sound classification and obtain a set of good practices that perform well in most of the current architectures and datasets.

### 3.1. Feature-Based Models for Audio

**Figure 1:**
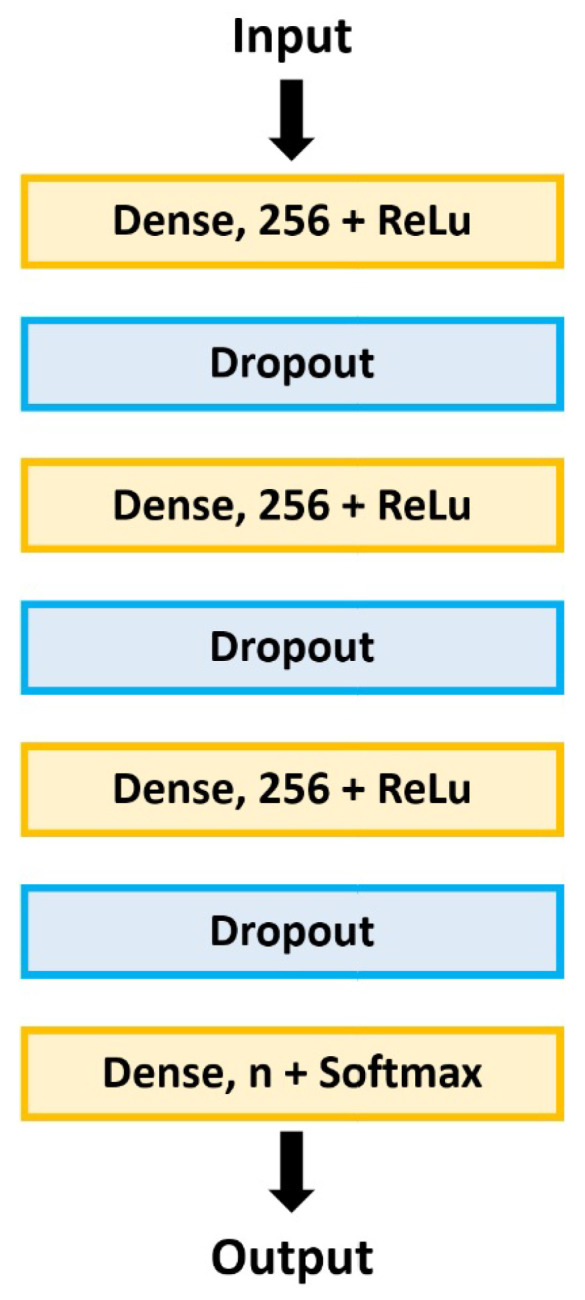
Baseline model’s architecture, where *n* is the number of classes.

The base model architecture consists of three fully connected layers with 256 units with ReLU as the activation function and one dense layer of 10 units using the Softmax function. Furthermore, there is a dropout layer between the fully connected layers. The scheme of this base model’s architecture is shown in Figure 1.

The fully connected layer has the neurons connected to every neuron of the preceding layer, making it deeply connected with its previous layer. The dropout layer randomly sets a percentage of the activations to 0 (zero) with a frequency rate at each step during training time. It scales up the other input values by 1/(1-rate) to the total sum remaining unchanged and prevent overfitting.

Different experiences were conducted to study the influence of the change rate on the dropout layer by employing different rate values, mainly [0, 0.2, 0.4, 0.6, and 0.8]. Besides improving the models’ performance, we also explored the increase of models’ depth by adding two extra layers: one fully connected layer and one dropout layer.

### 3.2. CNN for Audio

**Figure 2:**
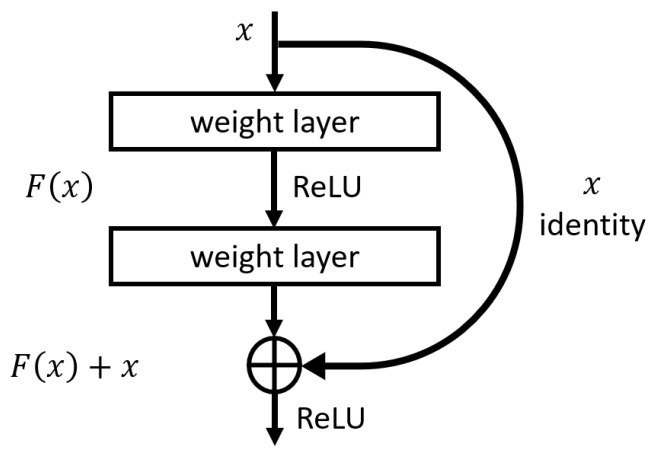
Residual block used in ResNet (adapted from He et al. [27]).

Residual Neural Network (ResNet) was introduced by He et al. [27] and uses a feedforward neural network with shortcut connections to solve the vanishing gradient problematic and mitigate the degradation of Deep Neural Network (DNN)s with several layers. Therefore, ResNet consists of a stack of residual blocks, an ensemble of convolutional layers followed by a batch normalization layer and a ReLU activation function with shortcut connections that skips a stack of layers and adds the input directly before the last ReLU activation function of the stack. Figure 2 represents a residual block. ResNet-50 was the model’s architecture used in this study.

**Figure 3:**
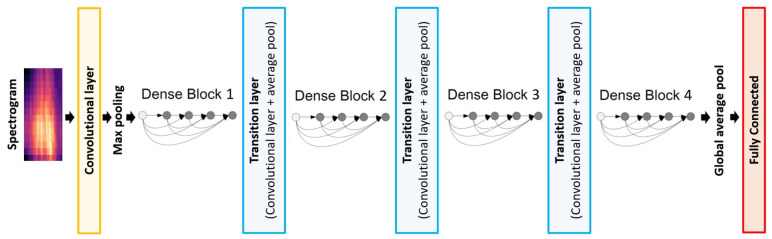
DenseNet model’s architecture.

Dense Convolutional Network (DenseNet) was introduced by Huang et al. [28] and used a dense connectivity pattern that directly connects all layers with matching feature map sizes to solve the vanishing gradient problem. Therefore, each layer obtains additional input from all preceding layers and passes on its feature maps to all subsequent layers. The final classifier can make decisions based on all feature maps in the network. The advantages of DenseNets are the flow of information and gradients throughout the network, the direct access that each layer has to the gradients from the loss function, and the original input signal facilitating the training of DCNN. DenseNet-201 was the network architecture used in this study, which has 6, 12, 48, and 32 convolutional layers in each dense block, respectively; see Figure 3.

Inception was developed by Szegedy et al. [29] to create a network that uses extra sparsity and exploits the current hardware by utilizing dense matrices to increase the DNNs’ depth and width, i.e., the number of units per level of depth, and consequently, improve the models’ performance. Therefore, the base ideas behind the Inception architecture were the need to find how an optimal local sparse structure in a convolutional network can be approximated and covered by readily available dense components. Furthermore, to judiciously apply dimension reduction and projection whenever, the computational requirements would otherwise increase too much. Thus, the network consists of several Inception layers, which are a combination of a 1 × 1 convolutional layer, a 3 × 3 convolutional layer, and a 5 × 5 convolutional layer with their output filter banks concatenated into a single output vector that serves as the input of the next layer, stacked upon each other with occasional max-pooling layers with a stride of 2. Besides, these layers should be used only at higher layers to make the model memory efficient during training, keeping the other convolutional layers. The main benefit of this architecture is that it makes it possible to increase the width of the network without uncontrollably increasing the computational complexity. However, if the architecture is scaled up, most computational gains can be immediately lost.

**Figure 4:**
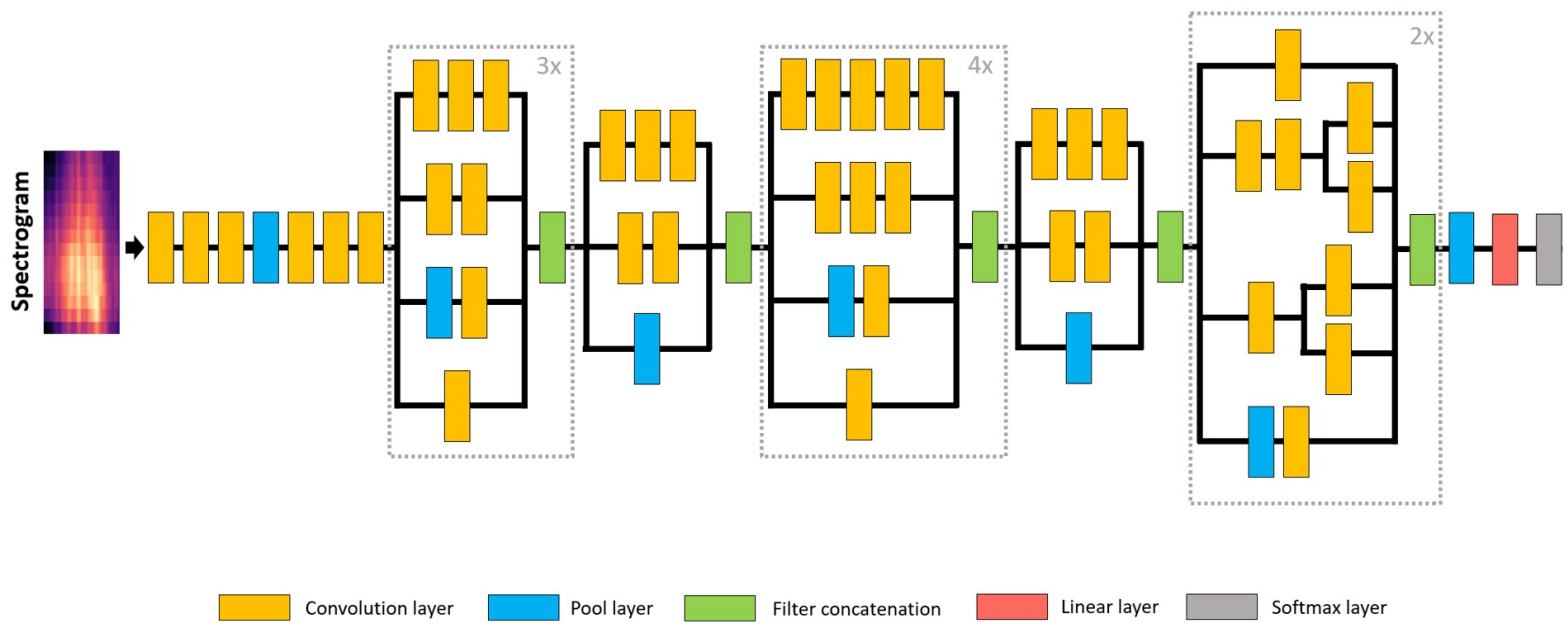
Inception-v3 model’s architecture.

Inception-v3, which was also developed by Szegedy et al. [30], was the architecture used in this work; see Figure 4.

### 3.3. Transformers for Audio

The Transformer model was proposed by Vaswani et al. [26] and consists of a transduction model that relies entirely on an attention mechanism to compute representations of its input and output. The model’s architecture consists of an encoder and a decoder. The encoder maps an input sequence of symbol representations to a sequence of continuous representations, and the decoder generates an output sequence of symbols one at a time. Furthermore, the model is auto-regressive at each step, because it uses the previously generated symbols as additional input when generating the next one.

**Figure 5:**
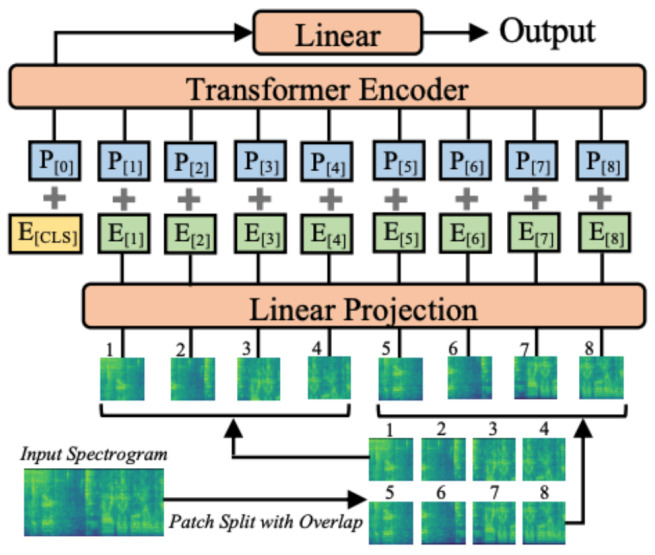
AST model’s architecture proposed by Gong et al. [9].

The architecture employed here is based on the AST model (Gong et al. [9]), which consists of a patch embedding layer that converts the input spectrogram into a sequence of patches and flattens it into a one-dimensional patch. Then, a trainable positional embedding is added to each patch embedding in order to capture the input order information and the temporal order of the patch sequence. In addition, a classification token is appended at the beginning of the sequence. The resulting sequence serves as the input for the standard Transformer’s encoder part. The encoder’s output of the classification token serves as the audio spectrogram representation, where a linear layer will map the labels using the sigmoid activation function for classification. Figure 5 illustrates the architecture of the model used in this study.

## 4. Experimental Validation

In the experimental validation, a set of defined hyperparameters were extensively evaluated among the considered models and the corresponding model’s architecture changed to obtain the best-performing model. Several datasets and metrics were used to assess the performance of the studied models.

### 4.1. Datasets

In this study, three significant datasets were used to conduct the experiments and evaluate the performance of the models under study according to the official splits by performing k-fold cross-validation. The UrbanSound8K, ESC-10, and ESC-50 datasets were selected due to being the most popular datasets used in Environmental Sound Classification (ESC). The given possibility is to examine the models’ behaviour when the number of classes is maintained, but the composition and name of the classes changes, and when there is an increase in the number of classes. The UrbanSound8K dataset contains 8732 audio samples from 10 urban sound classes not uniformly distributed with a maximum of 4 s; the ESC-10 dataset is formed by 2000 uniformly distributed samples from 10 classes, with 40 samples per class. The ESC-50 dataset is composed of 400 balanced samples from 50 audio classes, with 40 samples per class. The audio of both ESC-10 and ESC-50 datasets have 5 s of duration.

### 4.2. Experimental Setup and Baselines

To establish a working baseline to compare end-to-end CNN and Transformer model variations, the first step was to determine by extensive evaluation of the hyperparameters and architecture variation, a feature-based audio classification model that explores handcrafted audio features to discriminate the sound classes under study.

Baseline model: The baseline determination was achieved by several experiences that evaluated several potential variations of the baseline model (Figure 1), mainly the network depth, features to be used, dropout and learning rates, optimizers, and only attaining the top performing for each dataset. Thus, the final model was obtained after 100 epochs of training using Nadam optimizer, a dropout of 0.2 for the ESC datasets and of 0.6 for the UrbanSound8K dataset, a learning rate of 0.001, and two fully connected hidden layers with a size of 256.

CNN models: To determine the most advantageous end-to-end convolutional model, the ResNet, DenseNet, and Inception models had their performance evaluated with no pre-training, pre-training from ImageNet, and data augmentation using the combination of time stretch and pitch shift.

Transformer models: The best Transformer-based model was obtained by evaluating the effect of pre-training, in particular the difference between just distilling knowledge from an image domain model, ImageNet, or two models: one from the image domain, ImageNet, and the other from the audio domain, AudioSet. Furthermore, the influence of changing the batch size was explored, and finally, the differences that the various data augmentation techniques could give rise to were accessed.

### 4.3. Metrics

The performance of all models was evaluated using six different metrics: Accuracy, Area Under the Receiver Operating Characteristic (ROC) Curve (AUC), Precision, Recall, and the micro and macro F1-score, which was essential to compare them and select the best-performing model. All metric were implemented using the scikit-learn library.

**Accuracy** measures how often an algorithm classifies a data point correctly, so it is the number of correctly classified data points out of all data points. Mathematically, it can be defined as:

(1) $$\begin{array}{c} \mathrm{Accuracy}=\frac{n{}^{\circ}\mathrm{of}\;\mathrm{correct}\;\mathrm{predictions}}{\mathrm{Total}\;n{}^{\circ}\;\mathrm{of}\;\mathrm{predictions}}. \end{array}$$

**AUC** measures the ability of a classifier to distinguish between classes. It integrates the ROC curve graph that evaluates the model’s performance in all classification thresholds. Therefore, it measures how well predictions are ranked invariant to classification thresholds, measuring the quality of the model’s prediction independent of the chosen classification threshold.

**Precision** gives the number of correctly classified data points out of all points identified as being of a certain class. In binary classification, the problem can be defined as:

(2) $$\begin{array}{c} \mathrm{Precision}=\frac{TP}{TP+FP}, \end{array}$$

where TP is the number of true positives and FP is the number of false positives.

**Recall** gives the number of correctly classified data points out of all points belonging to the class on the dataset. In binary classification, the problem can be defined as:

(3) $$\begin{array}{c} \;\mathrm{Recall}=\frac{TP}{TP+FN}, \end{array}$$

where FN is the number of false negatives.

**F1-score** corresponds to the harmonic mean of Precision and Recall, which allows assessing model performance based on the values of two metrics:

(4) $$\begin{array}{c} F1\text{-}\mathrm{score}=\frac{2(\mathrm{Precision}*\mathrm{Recall})}{\mathrm{Precision}+\mathrm{Recall}}=\frac{TP}{TP+\frac{1}{2}\left(FP+FN\right)}. \end{array}$$

In multiclass problems, it is possible to define different averaging types to calculate the *n* F1-score metric. One is the micro average F1-score, which computes the global average score by summing all values across all classes for True Positive (TP)s, False Negative (FN)s, and False Positive (FP)s and then plugs it in the F1-score Equation (4). Furthermore, there is an F1-score calculated using an average macro scheme corresponding to the computation of the arithmetic mean of all instances per class F1-score.

### 4.4. Results and Discussion

With the conducted experiments on the different datasets used and variations of the models’ architecture, following an exhaustive hyperparameter tuning and refinement to fine-tune the models to obtain the best results, it was possible to observe that Transformers led to an excellent result in all datasets, surpassing the baseline by almost 36.9 percentage points (pp) in terms of accuracy, beating the end-to-end convolution models by a margin of 5.4 pp, on average. The aggregated results are presented in Table 1, and in Table 2 is indicated the required Graphics Processing Unit (GPU) memory capacity and computational time, which encompasses the training and inference time, aggregated by each dataset under study.

**Figure 6:**
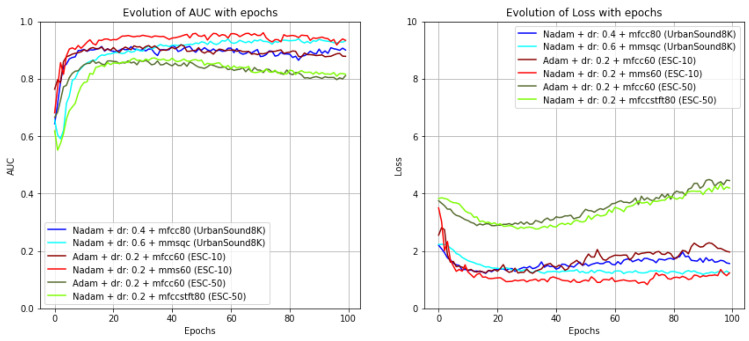
Graphs of the evolution of AUC (on the **left**) and loss function (on the **right**) along the epochs for the best baseline model for each used dataset with a single and a combination of features as the input (dr: dropout rate, mmsqc: MFCC + Mel spectrogram + Chroma STFT + Chroma CQT + Chroma CENS with 40 bins, mms60: MFCC + Mel spectrogram + Chroma STFT with 60 bins, mfccstft80: MFCC + Chroma STFT with 80 bins).

After a detailed results’ analysis, the following statements can be concluded for each of the model’s categories:

Baseline models: Out of the conducted experiments (Figure 6), the Nadam optimizer yielded the best results in most metrics for a single or a group of features extracted from the UrbanSound8K dataset, as well as for the ESC datasets, when features were used in combination. Concerning the input features, the single feature that provided better results in most metrics was MFCC, standing out especially: MFCC with 80 MFCCs for the UrbanSound8K dataset and with 60 MFCCs to the ESC datasets. Nonetheless, the results given by the models with a single feature input were surpassed when a group of features was used. The combination of MFCC, Mel spectrogram, Chroma Short-Term Fourier Transformation (STFT), Chroma Constant Q-Transform (CQT), and Chroma Chroma Energy Normalized Statistics (CENS) with 40 bins was the one that gave the best results in most metrics for the UrbanSound8K dataset and the second-best result out of all experiences for the ESC-10 dataset.

**Figure 7:**
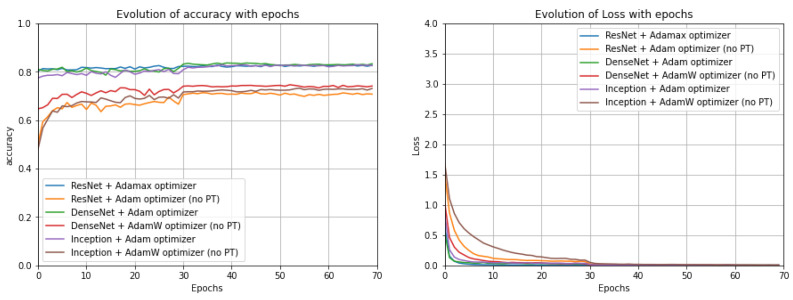
Evolution of the accuracy (on the **left**) and of the loss function (on the **right**) along the epochs for the studied models with the optimizer that led to the best results for each model for the UrbanSound8K dataset (no PT: not Pre-Trained).

**Figure 8:**
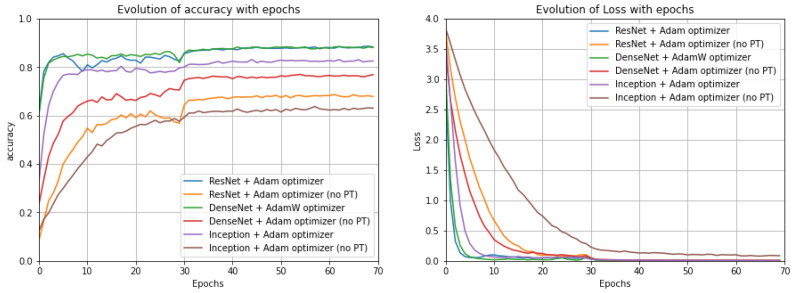
Evolution of the accuracy (on the **left**) and of the loss function (on the **right**) along the epochs for the studied models with the optimizer that led to the best results for each model for the ESC-50 dataset (no PT: not Pre-Trained).

**Figure 9:**
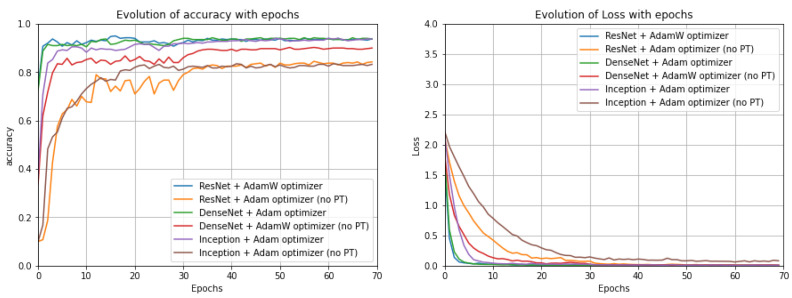
Evolution of the accuracy (on the **left**) and of the loss function (on the **right**) along the epochs for the studied models with the optimizer that led to the best results for each model for the ESC-10 dataset (no PT: not Pre-Trained).

CNN models: Analysing the results for the CNN pre-trained models with the UrbanSound8K dataset (Figure 7), it can be concluded that for the DenseNet and Inception models, Adam was the most beneficial optimization function; however, for ResNet, the best results were achieved when Adamax was chosen as the optimization function. Evaluating the performance of the best randomly initialized models, it was possible to conclude that, for all metrics, the pre-trained models provided better results with a difference of around 10 pp in most metrics. In addition, it was noticeable that the optimizer providing the best results for each architecture varied, and particularly for ResNet, Adam was the more suitable optimization function. On the other hand, for the DenseNet and Inception models, the AdamW optimization function gave the best results. Nonetheless, DenseNet gave the best results in most metrics for the randomly initialized models and in all metrics for the models with pre-trained model weights. For the ESC-50 dataset (Figure 8), for the ResNet and Inception models, the optimizer that provided the best results was Adam and for DenseNet was AdamW, which does not correspond to what was previously observed for the UrbanSound8K dataset. Regarding the ESC-10 dataset (Figure 9), Adam was the preferable optimization function for the DenseNet and Inception models; however, for ResNet, AdamW was the most beneficial one.

Out of all models for both ESC datasets, ResNet gave the best results in most metrics for the pre-trained models, but when no pre-training was used, once again, DenseNet was the more suitable model. It was also evident that the non-pre-trained models showed worse behaviour than the corresponding pre-trained models, with a difference of around 17 pp for the ESC-50 dataset and around 8 pp for the ESC-10 dataset, respectively.

The best pre-trained model for each dataset was used to test the influence of including data augmentation techniques in the training process, which was not beneficial in all cases. Nonetheless, for the ESC datasets, the best results were obtained when these techniques were employed, providing benefits ranging between 1 and 4 pp.

Transformers: Analysing the achieved results for the ESC datasets, it can be concluded that pre-training significantly impacted the results, leading to an improvement of around 43 pp in most used metrics between the no pre-trained and the pre-trained using ImageNet. However, these results were further improved by using both ImageNet and AudioSet pre-training with a difference of 7.2 pp for the ESC-50 dataset and of approximately 5.3 pp for the ESC-10 dataset in 4 out of the 6 metrics used, showing the importance of having models pre-trained in the same domain of the datasets under study.

**Figure 10:**
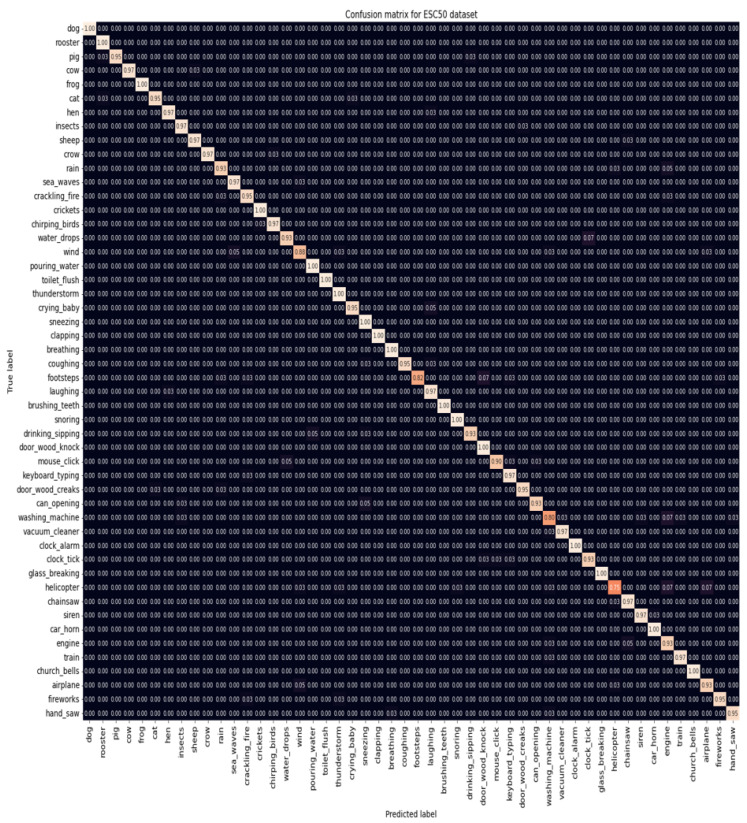
Confusion matrix for the ESC-50 dataset.

**Figure 11:**
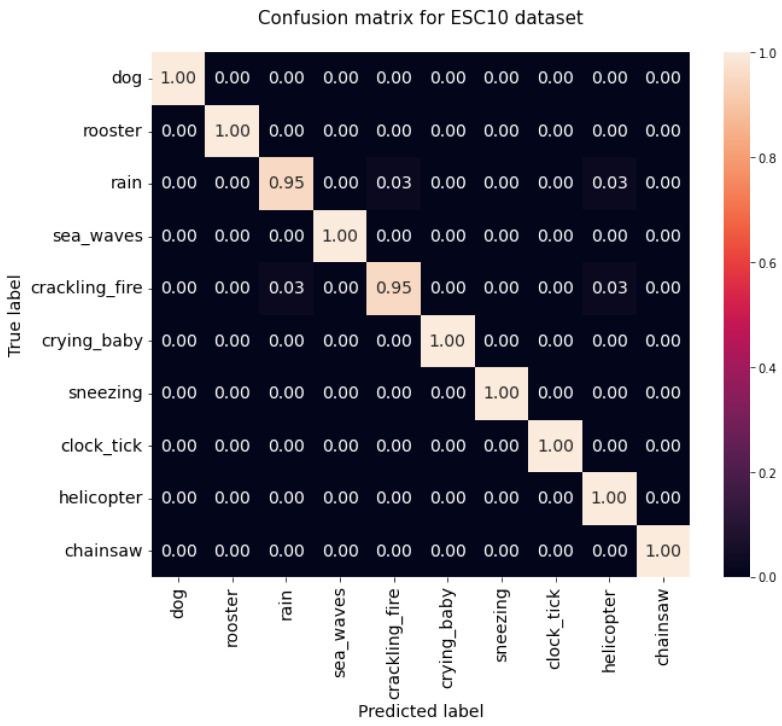
Confusion matrix for the ESC-10 dataset.

Out of all the conducted experiments, the best results were obtained with a Transformer pre-trained using ImageNet and AudioSet, a batch size of 48, and SpecAugment as the data augmentation. However, there was no consensus concerning the optimization function: AdamW for the ESC-50 dataset and Adam for the ESC-10 dataset were the most suitable ones. The corresponding confusion matrices revealed that, for the ESC-50 dataset (Figure 10), there were 17 classes with an accuracy of 100%, 29 with an accuracy equal to or superior to 90%, and only 4 classes with an accuracy inferior to 90%, which were washing machine, footsteps, wind, and helicopter sounds, with the lowest result being for the helicopter class with an accuracy of 75%. Concerning the ESC-10 dataset (Figure 11), rain and crackling fire were the only classes that did not achieve 100%. The sounds from the rain class were misclassified as belonging to the crackling fire and helicopter classes, and crackling fire sounds were misclassified as rain and helicopter. However, both classes achieved an accuracy of 95%.

**Figure 12:**
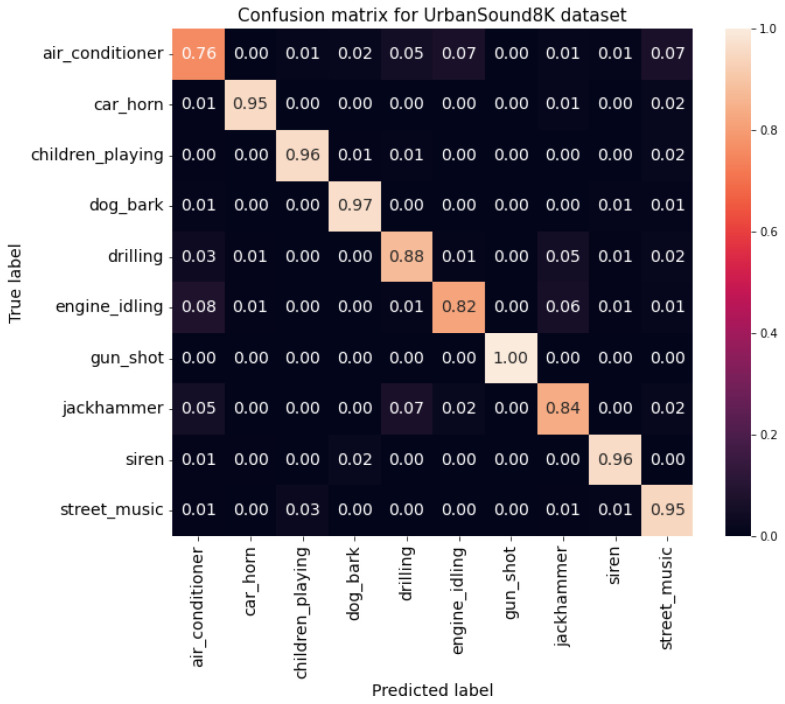
Confusion matrix for the UrbanSound8K dataset.

Using the best Transformer configuration, similar results were obtained for the UrbanSound8K dataset with the Adamax and AdamW optimization functions. However, Adamax was considered the preferable optimization function due to a more linear behaviour throughout the training epochs than AdamW. Furthermore, by analysing the confusion matrix shown in Figure 12, it is possible to identify that air conditioner, drilling, engine idling, and jackhammer were the most challenging classes. Nonetheless, besides all instances identified by the model, gunshot sounds belonged to the only class of sounds not mistaken for another class, and no class was confused as being of the gunshot class. Compared with the baseline and the CNN models, the improvement was, on average, of all metrics, of 51.4 pp and 4.95 pp for the ESC-50 dataset, of 21.0 pp and 3.40 pp for the ESC-10 dataset, and of 25.9 pp and 6.02 pp for the UrbanSound8K dataset, respectively.

Considering the results in Table 2, it is possible to conclude that, in terms of computational time, the baseline models were the fastest and did not require the use of a GPU; however, the models led to the poorest performance. As for the best pre-trained CNN model, even though they required less GPU memory capacity compared to the best Transformer model, the performance and computational time were far worse. These results confirm the superiority of the Transformer models, which can give highly reliable predictions relatively fast. The only downside is the required memory capacity.

## 5. Conclusions and Future Work

This study explored different CNN model variations, particularly DenseNet, ResNet, and Inception, with features learned from an input spectrogram and Transformer model variations, and compared them with different baseline models based on a simple set of dense layers. Several model parameters and strategies were evaluated: network depth, architecture, transfer learning from ImageNet and AudioSet, data augmentation strategies, fine-tuning hyperparameter, dropout rate, and optimization functions.

Pre-training based on ImageNet significantly increased the models’ performance regardless of the chosen architecture. Furthermore, using data augmentation techniques, depending on the used dataset or chosen architecture, might not always be advantageous due to the dramatic modification in sound characteristics that approximate one class to another. However, the SpecAugment technique proved to be the more effective of the studied models and datasets. Regarding the other experiments, the batch size was not a very significant parameter, apart from alleviating the memory footprint required to train the evaluated models. Concerning the various studied optimization functions, they dramatically depend on the model architecture, dataset, input, pre-training, and data augmentation used. These findings are in concordance with what the “no free lunch” theorem states. However, three optimization functions consistently gave the best performances: for the baseline models, the Nadam optimizer was the most suitable; for the end-to-end models, the Adam optimizer; for the Transformer, the best optimization function was AdamW for the ESC-50 dataset, Adam for the ESC-10 dataset, and Adamax for the UrbanSound8K dataset, respectively. This demonstrates that, although no optimization function is equally good for all situations, it is possible to find one more adequate for each circumstance, which reaffirms the “no free lunch theorem”.

However, there was a common difficulty between all the models concerning the accuracy per class for the UrbanSound8K dataset. All models exhibited lower accuracy values in the air conditioner, engine idling, jackhammer, and drilling classes, which must be similar because they were mostly misclassified as being of each other’s classes. In addition, having the gunshot class as the most accessible class was also a common point among all studied models. Regarding the ESC datasets, the most challenging class was the helicopter class, which presented the low scores in most situations. The most straightforward class was sneezing for the ESC-10 dataset and toilet flush for the ESC-50 dataset.

As a final model overview, Transformer was shown to be the most capable of providing better results by offering significant improvements compared with the best baseline and end-to-end model for each studied dataset, with an average difference between all metrics of 32.8 pp and 4.79 pp, respectively. In this work, it was not possible to achieve state-of-the-art results. However, very competitive results were obtained by providing the second-best result on the ESC-10 dataset with only a difference of 0.22 pp to the top outcome, the third-best score for the ESC-50 dataset, and the fourth-best for UrbanSound8K considering the official splits.

The differences between the baseline and the other models were not only due to the less complex architecture, but also due to the inferiority of the handcrafted features compared to deep features to produce more distinguishable representations of the different sounds. The superiority of the Transformer models compared with the other implemented models can be explained by the attention mechanisms focusing more on the most important distinguishable parts of the sound.

For future work, mainly to improve the model’s robustness, the introduction of new augmentation techniques should be taken into account, such as Patchout or random erase.

## Figures and Tables

**Table 1:** Summary and discussion of the studied models.

Model	DA	PT-I	PT-A	Metrics	Discussion
Dataset: ESC-10					
Baseline model + mms60 + Nadam + dr: 0.2	-	-	-	acc: 74.8%, AUC: 94.8%, mf1: 74.8%, Mf1: 74.3%, prec: 77.7%, rec: 73.3%.	The combination of features gives more discriminating information to the baseline model.
DenseNet + AdamW	-	-	-	acc: 89.8%, AUC: 98.9%, mf1: 89.8%, Mf1: 89.3%, prec: 91.5%, rec: 89.8%.	Improves the baseline performance by 13.23 pp. on average.
ResNet + AdamW	-	✓	-	acc: 94.0%, AUC: 99.8%, mf1: 94.0%, Mf1: 93.8%, prec: 94.8%, rec: 94.0%.	The use of pre-training from ImageNet improves, on average, the end-to-end model performance by 3.55 pp.
DenseNet + Adam	✓	✓	-	acc: 95.0%, AUC: 99.8%, mf1: 95.0%, Mf1: 94.9%, prec: 95.8%, rec: 95.0%.	The addition of data augmentation techniques provides a slight improvement of 0.85 pp, on average.
Transformer + AdamW	✓	-	-	acc: 52.8%, AUC: 91.8%, mf1: 52.8%, Mf1: 50.7%, prec: 61.7%, rec: 52.8%.	The use of a Transformer model without pre-training cannot give competitive results.
Transformer + AdamW	✓	✓	-	acc: 93.8%, AUC: 99.8%, mf1: 93.8%, Mf1: 93.5%, prec: 98.2%, rec: 93.8%.	The use of pre-training from ImageNet gives the Transformer model an average boost of 35.05 pp, showing the need for large datasets to train.
Transformer + AdamW	-	✓	✓	acc: 98.8%, AUC: 100%, mf1: 98.8%, Mf1: 98.7%, prec: 99.7%, rec: 98.8%.	Using pre-training from ImageNet and AudioSet gives a better performance than just an ImageNet pre-trained Transformer with an average increase of 3.65 pp.
Transformer + Adam	✓	✓	✓	acc: 99.0%, AUC: 100%, mf1: 99.0%, Mf1: 99.0%, prec: 99.9%, rec: 99.0%.	The addition of data augmentation to the pre-trained network from both domains gives, on average, a slight improvement of 0.18 pp. The average boost for the baseline model is 21.03 pp and for the best end-to-end model is 3.40 pp.
Dataset: ESC-50					
Baseline model + mfccstft80 + Nadam + dr: 0.2	-	-	-	acc: 38.1%, AUC: 82.4%, mf1: 38.1%, Mf1: 36.2%, prec: 43.9%, rec: 33.9%.	The combination of features gives more discriminating information to the baseline model.
DenseNet + Adam	-	-	-	acc: 76.1%, AUC: 98.7%, mf1: 76.1%, Mf1: 75.4%, prec: 78.0%, rec: 76.1%.	Improves the baseline performance by 34.63 pp, on average.
ResNet + Adam	-	✓	-	acc: 88.2%, AUC: 99.6%, mf1: 88.2%, Mf1: 87.7%, prec: 89.6%, rec: 88.2%.	The use of pre-training from ImageNet improves, on average, the end-to-end model performance by 10.18 pp.
ResNet + Adam	✓	✓	-	acc: 90.1%, AUC: 99.6%, mf1: 90.1%, Mf1: 89.9%, prec: 91.2%, rec: 90.1%.	The addition of data augmentation techniques gives a small increase of 1.58 pp, on average.
Transformer + AdamW	✓	-	-	acc: 43.9%, AUC: 93.6%, mf1: 43.9%, Mf1: 42.4%, prec: 46.8%, rec: 43.9%.	The use of a Transformer model without pre-training is not capable of giving good results; however, they are better than the baseline model.
Transformer + AdamW	✓	✓	-	acc: 88.6%, AUC: 99.6%, mf1: 88.6%, Mf1: 88.4%, prec: 92.7%, rec: 88.6%.	The use of pre-training from ImageNet gives a huge performance boost of 38.67 pp, on average, compared to the Transformer model without pre-training.
Transformer + AdamW	-	✓	✓	acc: 95.4%, AUC: 99.9%, mf1: 95.4%, Mf1: 95.3%, prec: 97.6%, rec: 95.4%.	Using pre-training from ImageNet and AudioSet gives an average improvement of 5.42 pp compared to the ImageNet pre-trained Transformer.
Transformer + AdamW	✓	✓	✓	acc: 95.8%, AUC: 99.9%, mf1: 95.8%, Mf1: 95.6%, prec: 97.8%, rec: 95.8%.	The addition of data augmentation gives a small improvement of 0.28 pp, on average. The average boost is for the baseline model of 51.35 pp and of 4.95 pp for the best end-to-end model.
Dataset: UrbanSound8K					
Baseline model + mmsqc + Nadam + dr: 0.6	-	-	-	acc: 61.1%, AUC: 88.9%, mf1: 61.1%, Mf1: 63.2%, prec: 73.1%, rec: 49.2%.	The combination of features gives more discriminating information to the baseline model.
DenseNet + AdamW	-	-	-	acc: 74.2%, AUC: 95.4%, mf1: 74.2%, Mf1: 75.6%, prec: 75.2%, rec: 74.2%.	Improves the baseline performance by 12.03 pp, on average.
DenseNet + Adam	-	✓	-	acc: 83.3%, AUC: 97.7%, mf1: 83.3%, Mf1: 84.4%, prec: 84.1%, rec: 83.3%.	The use of pre-training from ImageNet improves the end-to-end model performance by 7.88 pp, on average.
ResNet + Adamax	✓	✓	-	acc: 82.2%, AUC: 97.4%, mf1: 82.2%, Mf1: 83.0%, prec: 82.5%, rec: 82.2%.	The use of data augmentation techniques was detrimental.
Transformer + Adamax	✓	✓	✓	acc: 89.8%, AUC: 98.6%, mf1: 89.8%, Mf1: 90.4%, prec: 93.8%, rec: 89.8%.	The Transformer model pre-trained with datasets from both domains and using data augmentation gives an average boost of 25.93 pp regarding the baseline model and of 6.02 pp compared to the best end-to-end model.

DA: Data Augmentation; PT-I: Pre-Trained ImageNet; PT-A: Pre-Trained AudioSet; dr: dropout rate; acc: accuracy; AUC: Area Under the receiver operating characteristic Curve; mf1: micro F1-score; Mf1: macro F1-score; prec: precision; rec: recall; pp: percentage points; mmsqc: MFCC + Mel spectrogram + Chroma STFT + Chroma CQT + Chroma CENS with 40 bins; mms60: MFCC + Mel spectrogram + Chroma STFT with 60 bins; mfccstft80: MFCC + Chroma STFT with 80 bins.

**Table 2:** GPU capacity and computational time required by the studied models.

Model	DA	PT-I	PT-A	GPU Capacity (MiB)	Computational Time (min)
Dataset: ESC-10					
Baseline model + mms60 + Nadam + dr: 0.2	-	-	-	-	8
DenseNet + AdamW	-	-	-	5913	29
ResNet + AdamW	-	✓	-	3591	18
DenseNet + Adam	✓	✓	-	5913	907
Transformer + AdamW	✓	-	-	39,444	32
Transformer + AdamW	✓	✓	-	39,444	37
Transformer + AdamW	-	✓	✓	39,444	34
Transformer + Adam	✓	✓	✓	39,444	32
Dataset: ESC-50					
Baseline model + mfccstft80 + Nadam + dr: 0.2	-	-	-	-	25
DenseNet + Adam	-	-	-	5904	130
ResNet + Adam	-	✓	-	3566	66
ResNet + Adam	✓	✓	-	3566	886
Transformer + AdamW	✓	-	-	39,712	74
Transformer + AdamW	✓	✓	-	39,712	64
Transformer + AdamW	-	✓	✓	39,712	68
Transformer + AdamW	✓	✓	✓	39,712	65
Dataset: UrbanSound8K					
Baseline model + mmsqc + Nadam + dr: 0.6	-	-	-	-	46
DenseNet + AdamW	-	-	-	5902	762
DenseNet + Adam	-	✓	-	5902	733
ResNet + Adamax	✓	✓	-	3564	2541
Transformer + Adamax	✓	✓	✓	39,716	370

DA: Data Augmentation; PT-I: Pre-Trained ImageNet; PT-A: Pre-Trained AudioSet; dr: dropout rate; mmsqc: MFCC + Mel spectrogram + Chroma STFT + Chroma CQT + Chroma CENS with 40 bins; mms60: MFCC + Mel spectrogram + Chroma STFT with 60 bins; mfccstft80: MFCC + Chroma STFT with 80 bins.

## Data Availability

Not applicable.

## Abbreviations

The following abbreviations are used in this manuscript:

AAML	Additive Angular Margin Loss
AST	Audio Spectrogram Transformer
AUC	Area Under the Receiver Operating Characteristic (ROC) Curve
BERT	Bidirectional Encoder Representations from Transformers
CENS	Chroma Energy Normalized Statistics
CNN	Convolutional Neural Networks
CQT	Constant Q-Transform
CRNN	Convolutional Recurrent Neural Networks
DCNN	Deep Convolutional Neural Networks
DenseNet	Dense Convolutional Network
DL	Deep Learning
DNN	Deep Neural Network
ESC	Environmental Sound Classification
FN	False Negative
FP	False Positive
GPU	Graphics Processing Unit
LSTM	Long Short-Term Memory
M2M-AST	Many-to-Many Audio Spectrogram Transformer
MFCC	Mel Frequency Cepstral Coefficients
NLP	Natural Language Processing
pp	percentage points
ResNet	Residual Neural Network
RNN	Recurrent Neural Networks
STFT	Short-Term Fourier Transformation
TFCNN	Temporal-Frequency attention-based Convolutional Neural Network
TP	True Positive
VATT	Video–Audio–Text Transformer
